# Amyotrophic Lateral Sclerosis and Myasthenia Gravis Overlap Syndrome: A Review of Two Cases and the Associated Literature

**DOI:** 10.3389/fneur.2017.00218

**Published:** 2017-05-22

**Authors:** Hongfei Tai, Liying Cui, Yuzhou Guan, Mingsheng Liu, Xiaoguang Li, Yan Huang, Jing Yuan, Dongchao Shen, Dawei Li, Feifei Zhai

**Affiliations:** ^1^Department of Neurology, Peking Union Medical College Hospital, Peking Union Medical College, Chinese Academy of Medical Sciences, Beijing, China; ^2^Neuroscience Center, Chinese Academy of Medical Sciences, Beijing, China

**Keywords:** amyotrophic lateral sclerosis, myasthenia gravis, overlap, clinical characteristics, immunological mechanism

## Abstract

**Objective:**

To describe the characteristics of patients with amyotrophic lateral sclerosis (ALS) and myasthenia gravis (MG) overlap syndrome and explore the relationship between the two diseases.

**Methods:**

We conducted a search of medical records at Peking Union Medical University Hospital from 1983 to 2015 for coexistence of ALS and MG and searched the PubMed database for all literature describing ALS and MG overlap syndrome published through December 2016. We analyzed the clinical and neurophysiological characteristics of patients by groups according to strict diagnostic criteria.

**Results:**

We presented 2 patients in our database with combined ALS and MG, and together with 25 cases reported in the literature, the patients were divided into 4 groups: 12 patients with MG followed by ALS, 8 patients with ALS followed by MG, 5 ALS patients with false-positive anti-acetylcholine receptor, and the other 2 ALS patients with only myasthenia symptoms. Most patients had limb onset ALS, and myasthenia symptoms mainly affected ocular and bulbar muscles. Clinical and neurophysiological characteristics were summarized.

**Conclusion:**

These findings support the conclusion that immunological mechanisms and alterations in the neuromuscular junction are related to ALS pathogenesis.

## Introduction

Amyotrophic lateral sclerosis (ALS) and myasthenia gravis (MG) are different disorders affecting motor neurons and neuromuscular junctions, respectively. ALS is a progressive neurodegenerative disorder involving primarily motor neurons in the cerebral cortex, brain stem, and spinal cord, and it is characterized by muscle weakness and muscular atrophy (1). The pathogenesis of ALS is not well understood, resulting in a lack of appropriate therapy. By contrast, MG is a disorder of neuromuscular transmission, resulting from binding of autoantibodies to components of the neuromuscular junction, characterized by muscle weakness and fatigability (2). The most common antibody is against the acetylcholine receptor (AChR), and other targets, such as the muscle-specific kinase (MuSK) protein or lipoprotein-related protein 4 (LRP4), have been described (3). Treatment includes immune-regulating therapy and symptomatic therapy, resulting in good prognosis for most patients. Coexistence of ALS and MG has been described in a few reports. This coexistence is considered to be far beyond coincidence (4) and suggests a relationship between MG and ALS involving immunological mechanisms. In this study, we described two cases of our own and reviewed the cases previously published in English in detail.

## Materials and Methods

### Retrospective Study of Our Database

We searched all medical records at Peking Union Medical University Hospital (PUMCH) from January 1983 to December 2015 for International Classification of Diseases (ICD)-9 and ICD-10 diagnostic codes for coexistence of MG and ALS. We retrospectively re-evaluated the diagnoses based on clinical presentations and neurophysiological examinations, then followed up with patients by telephone.

### Search of the Literature

PubMed, Embase, and Chinese biomedical databases were searched for papers published up to December 2016 that reported coexistence of ALS and MG. Search terms included (“amyotrophic lateral sclerosis” or “motor neuron disease” or “ALS” or “MND”) AND (“myasthenia gravis” or “myasthenia” or “MG”). Both text word and MeSH subject headings were used. The search strategy was supplemented by inspecting the reference lists of the included articles. Language was confined to English and Chinese. The papers were considered for inclusion if they described patients with coexistence of ALS and MG and provided diagnostic evidence. ALS was diagnosed according to the revised El Escorial World Federation of Neurology criteria (1). The diagnosis of MG was made based on clinical and accessory findings. Suggestive clinical features were fluctuating muscle weakness with exacerbation by exercise as well as improvement after cholinesterase inhibitor treatment. Laboratory tests supporting the diagnosis of MG were detection of specific autoantibody and a significant decrement in low-frequency repetitive nerve stimulation (RNS) test. Studies were excluded if they (1) reported coexistence of MG and other forms of neuronopathy, including spinal muscular atrophy and Kennedy’s disease (2); diagnosed either ALS or MG without sufficient evidence (3); and were epidemiological studies without sufficient patient details.

The clinical characteristics of each patient, including gender, nationality, interval between onset of the two diseases, clinical presentations, accessory exams, therapy, and prognosis, were extracted from the included studies. They were reviewed, and summarized and placed into groups according to strict diagnostic criteria.

## Results

### Case Presentation

Three cases were found from PUMCH medical records listed with diagnoses of both ALS and MG. Two of the cases were combined ALS and MG, and the other one was finally confirmed to be ALS that was misdiagnosed as MG at an early stage (not shown).

#### Case 1

One patient in his 40s presented with progressive left upper limb weakness with muscle atrophy for 8 months. Three months before consultation, fluctuating diplopia and ptosis appeared, along with paroxysmal palpitation, mild right hand tremor, irritability, chronic diarrhea, and a 5-kg weight loss. His right arm was also affected with mild weakness within the previous 3 weeks. On examination, bilateral lid paresis, ophthalmoplegia, and diplopia were noted, and the patient had a positive ptosis fatigue test. There was severe muscle atrophy in the left arm and both hands. The muscle strength of the patient’s upper limbs was 3/5 on MRC. Though manual muscle tests of the lower limbs were normal, fatigable weakness could be induced after exercise. The patient had brisk deep tendon reflexes in the lower limbs, bilaterally positive Hoffmann’s signs and Babinski’s signs. Electromyogram (EMG) revealed generalized motor neuron lesion with signs of both acute and chronic denervation. Significant decrement (up to 38%) was seen after RNS test on the left axillary nerve. A neostigmine test was positive. AChR antibodies (AChR-ab) were normal in concentration. A computed tomography (CT) scan of mediastinum showed thymic hyperplasia. The patient was also found to have hyperthyroidism and was prescribed Thyrozol. His ocular symptoms and fatigable weakness improved after treatment with intravenous immunoglobulin (IVIG) at a dose of 0.4 g/kg for 5 days, with sequential oral prednisone and pyridostigmine. However, limb weakness worsened irreversibly, and dysphagia, dysarthria, and dyspnea appeared during the following year.

#### Case 2

A patient in his 50s was diagnosed with ocular MG due to fluctuating bilateral ptosis and diplopia. The symptoms went into full remission after treatment with 60 mg QD pyridostigmine bromide. The patient was doing well without medication until 5 years later, when the ocular symptoms were aggravated, and he developed muscle weakness and wasting in bilateral lower limbs, progressively affecting all extremities and the bulbar region within 6 months. Pyridostigmine bromide (60 mg TID) could partially improve the ocular symptoms, but it could not be tolerated because of generalized fasciculation. Upon examination, he had bilateral ptosis, restricted eye movements and fasciculations in the tongue. Generalized brisk tendon reflexes, bilateral ankle clonus, Hoffmann’s sign, flexor plantar response, and spastic gait suggested upper motor neuronal damage. EMG showed fibrillation potentials and positive sharp waves together with signs of chronic denervation in all extremities, the paraspinal muscles, and the sternocleidomastoid. AChR-ab was 39.06 nmol/l (normal <0.4 nmol/l). RNS, thyroid function, and CT scan of the mediastinum were all normal. He was given 0.4 g/kg IVIG for 5 days, causing complete resolution of ocular symptoms, but the treatment had no effect on the general weakness. He was diagnosed with clinically probable ALS according to the revised El Escorial World Federation of Neurology criteria (1), and riluzole was prescribed. Respiratory function was eventually involved, and the patient died 28 months after ALS onset.

### Characteristics of ALS and MG Overlap Syndrome

**Figure 1:**
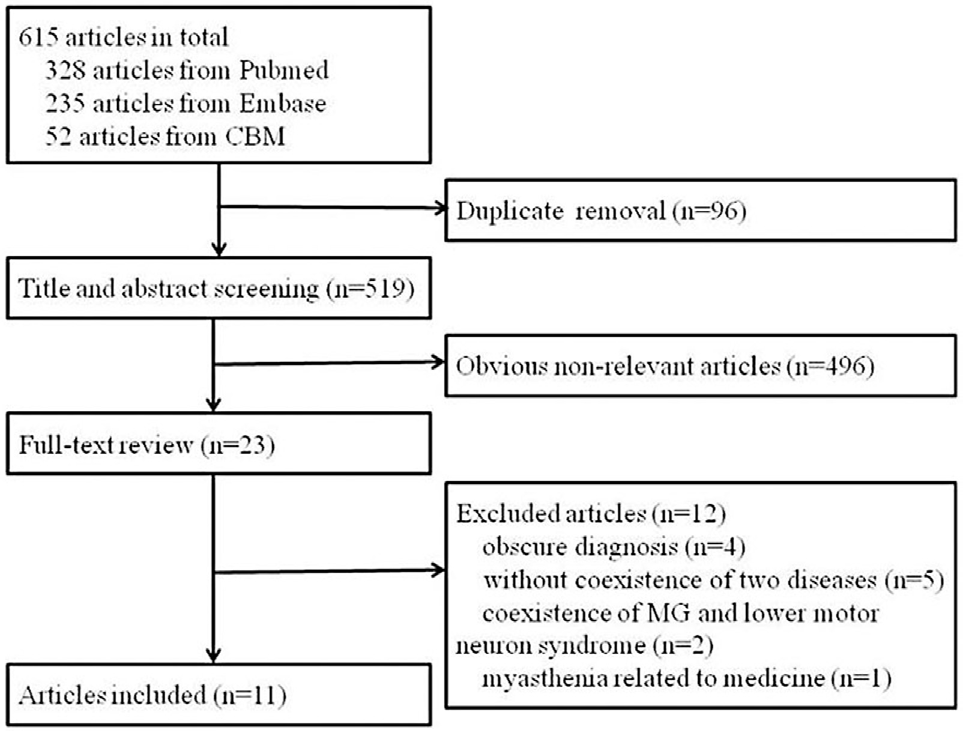
**Flow chart of article retrieval**.

A total of 615 articles were identified. After removing duplicate articles, the 519 remaining articles were screened by title and abstract. A total of 23 full-text articles were assessed for eligibility. Four studies had an obscure diagnosis of either ALS or MG or lack of important information (5–8), five differentiated the two diseases but not their coexistence (9–13), two reported coexistence of MG and lower motor neuron syndrome (14, 15), and one described an ALS patient who developed myasthenia possibly due to the use of riluzole (16). Finally, 11 articles that reported cases diagnosed as coexistence of ALS and MG were identified (4, 17–26). A flow chart of the method of article retrieval is presented in Figure 1.

Altogether, the characteristics of 25 cases reported in the literature and 2 cases of our own were reviewed. According to their clinical course and presentation, the cases could be roughly divided into four groups (Tables 1–3 for groups 1–3, respectively).

**Table 1:** **Patients with MG as inaugural disease followed by ALS**.

Patients	Nationality	MG classification	Localization of MG symptoms	Abnormal fatigability	Neostigmine test or response to therapy	AChR-ab (nmol/l)	RNS CMAP decrement (%)	CT scan of mediastinum	Associated immune-mediated disease
1 (20)	Indian	Ocular MG	Ocular	+	+	8.04 (*N* < 0.04)	19–33	Normal	No
2 (21)	Japanese	Generalized MG	Ocular, bulbar, UL, LL	+	+	3.8	>10	Thymectomy	No
3 (22)	French	Generalized MG	Ocular, head drop	+	+	40 (*N* < 0.5)	>10	Normal	No
4 (22)	French	Generalized MG	Bulbar	NA	+	8.4 (*N* < 0.5)	>10	Thymectomy	No
5 (22)	French	Generalized MG	Bulbar	+	+	>8 (*N* < 0.5)	NA	NA	Basedow’s disease
6—case 2	Chinese	Ocular MG	Ocular	+	+	39.06 (*N* < 0.4)	5	Normal	No
7 (4)	Italian	Generalized MG	Ocular, bulbar, LL	+	+	Positive	27	Normal	No
8 (4)	Italian	Ocular MG	Ocular	NA	+	Positive	21	Thymoma	No
9 (4)	Italian	Ocular MG	Ocular	+	+	Negative	12	Normal	No
10 (4)	Italian	Ocular MG	Ocular	+	+	Negative	ND	Normal	Hyperthyroidism
11 (4)	Italian	Generalized MG	Ocular, bulbar, UL, LL	+	+	Negative	20	Normal	No
12 (25)	Israel	Generalized MG	Ocular, bulbar, limbs	+	+	Positive	NA	Normal	Thyroiditis, ANA(+)

**No. patients**	**Interval between two conditions**	**ALS classification**	**Site of onset of ALS**	**Regions of EMG neurogenic pattern**	**MG relapsed while ALS onset**	**Immune-modulating therapy after ALS onset**	**Effects on myasthenia/ALS symptoms**	**Riluzole**	**Prognosis (worsened or ALS survival)**

1 (20)	38 years	Definite	UL + LL	UL/LL/bulbar/paraspinal	Yes	IVIG	Transient effect	No	Worsened
2 (21)	41 years	Probable	Bulbar	UL/LL/bulbar	No	Prednisolone, tacrolimus	−/no effect	Yes	Died/3 years
3 (22)	11 months	Probable	UL proximal	UL/LL/bulbar	No	IVIG, plasmapheresis	−/no effect	No	Died/26 months
4 (22)	319 months	Probable-lab supported	UL proximal	UL/LL	No	IVIG, corticosteroids	−/partial improvement during the first 6 months	Yes	Died/2 years
5 (22)	8 months	Probable-lab supported	LL distal	UL/LL/bulbar	No	IVIG, corticosteroids	−/no effect	Yes	Died/6 years
6—case 2	5 years	Probable	LL proximal	UL/LL/bulbar/paraspinal	Yes	IVIG	Complete resolution/no effect	No	Died/28 months
7 (4)	3 months	Probable	LL	NA	No	NA	NA	NA	Died/6 months
8 (4)	2 years	Probable	UL + LL distal	NA	No	NA	NA	NA	Died/12 months
9 (4)	1 years	Definite	Bulbar	NA	No	NA	NA	NA	Respiratory failure after 12 months
10 (4)	22 years	Probable	Bulbar	NA	No	NA	NA	NA	Respiratory failure after 12 months
11 (4)	6 months	Definite	Bulbar	NA	No	NA	NA	NA	Alive after 12 months
12 (25)	2 years	Definite ALS–FTD	Limbs	UL/LL	No	Prednisolone, azathioprine, MSC	Both improved	No	Transient improved

*M, male; F, female; UL, upper limb; LL, lower limb; NA, not applied; CMAP, compound muscle action potential; CT, computed tomography; EMG, electromyogram; IVIG, intravenous immunoglobulin; ALS–FTD, amyotrophic lateral sclerosis–frontotemporal dementia; MSC, mesenchymal stem cells; ND, not done; MG, myasthenia gravis; AChR-ab, acetylcholine receptor antibodies; RNS, repetitive nerve stimulation*.

Group 1 included patients diagnosed with MG as the inaugural disease followed by ALS (details in Tables 1 and 4). There were 12 patients (male:female = 8:4) who had been diagnosed with MG 3 months to 41 years before they developed ALS. Although the onset of MG occurred at different ages (26–82 years old) and mainly affected ocular and bulbar muscles, they developed ALS at a mean age of 71 years old (55–83 years old) with limb or bulbar onset (limb:bulbar = 8:4). Comparing the localization of myasthenia symptoms and the site of onset of ALS for these patients, they were always in different regions except in three cases. Myasthenia symptoms in two patients relapsed or were aggravated concomitant with ALS symptoms, but they were sensitive to IVIG treatment. The mean survival time of seven cases was approximately 29 months.

Group 2 contained patients diagnosed with ALS as the inaugural disease followed by MG (details in Tables 2 and 4), including eight patients (male:female = 6:2). There was a profound discrepancy between their ages at onset of ALS (34–89 years old). However, most patients developed MG within the following 18 months except one. Four patients developed the two diseases almost at the same time, and it was difficult to distinguish the order of onset. As in Group 1, most cases (5/7) were limb onset ALS, and myasthenia symptoms only affected ocular and bulbar muscles. In addition to the most common AChR-ab associated MG in six patients, the other two patients were positive for LRP4-ab (24). Myasthenia symptoms could be improved or relieved through immune-modulating therapy, and some recovered spontaneously. It seemed that ALS in younger patients progressed more slowly (22).

**Table 2:** **Patients with ALS as inaugural disease followed by MG**.

Patients	Nationality	ALS classification	Site of onset of ALS	Regions of EMG neurogenic pattern	Interval time between two disease	Localization of myasthenia symptoms	Neostigmine test	AChR-ab (nmol/l)	RNS CMAP decrement (%)	CT scan of mediastinum	Associated immune-mediated disease	Immune-directed therapy	Effect on myasthenia symptoms	Riluzole	Prognosis (worsened or Died/survival)
1 (22)	French	Definite	UL distal	UL/LL/bulbar	5 years	Ocular, head drop	+	AChR-ab 5.9 (*N* < 0.5)	>10	Normal	No	No	Improve	Yes	Stable in 1 year
2 (22)	French	Probable	Bulbar	UL/LL	18 months	Ocular	+	AChR-ab 10 (*N* < 2)	12–16	Normal	No	No	Spontaneous remission	Yes	Progress very slowly in 15 years
3 (22)	French	Probable	LL distal	UL/LL	6 months	Bulbar, head drop	+	AChR-ab > 100 (*N* < 0.5)	13–50	NA	No	IVIG, corticosteroids	Completely recovered	Yes	Worsened quickly
4—Case 1	Chinese	Probable-lab supported	UL proximal	UL/LL/bulbar/paraspinal	5 months	Ocular	+	AChR-ab 1.0 (*N* < 1.1)	38	Thymic hyperplasia	Hyperthyroidism	Corticosteroids, IVIG	Significantly improved	No	Worsened slowly
5 (23)	Norway	Probable	Limb	UL/LL/bulbar	0	Ocular	+	AChR-ab 16	40	Normal	No	Plasmapheresis, corticosteroid, azathioprine	Improved	NA	Died/16 mo
6 (26)	Caucasian	Definite	NA	NA	0	Ocular, bulbar	NA	Positive	>10	NA	No	IVIG, prednisone	Improved	NA	Worsened
7 (24)	Japanese	Probable	Bulbar	UL/LL/bulbar	0	Ocular, bulbar	+	Anti-LRP4 1.08 (*N* < 1.0)	Normal	Normal	No	Steroid, IVIG, plasmapheresis	Improved partially	NA	Worsened
8 (24)	Japanese	Probable	UL distal	UL/LL/bulbar/paraspinal	0	Ocular, head drop	+	Anti-LRP4 1.5 (*N* < 1.0)	10.6	NA	No	Steroid, plasmapheresis	Improved	NA	Died/16 months

*UL, upper limb, LL, lower limb, NA, not applied, CMAP, compound muscle action potential, EMG, electromyogram, IVIG, intravenous immunoglobulin; ALS, amyotrophic lateral sclerosis; MG, myasthenia gravis; AChR-ab, acetylcholine receptor antibodies; RNS, repetitive nerve stimulation; CT, computed tomography*.

Group 3 contained five ALS patients (female:male = 3:2; age at onset of ALS: 37–88 years old) who were only positive for AChR-ab in sera (Table 3) but did not have any other characteristics of MG, including fluctuating symptoms, abnormal RNS test, or response to cholinesterase inhibitors. We characterize these patients as having ALS with false-positive AChR-ab as in previous papers (17).

**Table 3:** **ALS patients with false-positive AChR-ab**.

Patients	Nationality	ALS classification	EMG	Fluctuating symptoms	AChR-ab (nmol/l)	RNS CMAP decrement (%)	CT scan of mediastinum	Effect of cholinesterase inhibitor	Associated immune-mediated disease
1 (17)	Japanese	Possible	LL	No	0.5 (*N* < 0.2)	0	Normal	Not use	No
2 (19)	American	Probable-lab supported	Generalized	No	1.64–19 (*N* < 0.4)	Normal	Normal	Never use	No
3 (22)	French	Probable	UL/LL/bulbar	No	2.4 (*N* < 0.5)	NA	NA	No effect	No
4 (23)	Norway	Probable	UL/LL/diaphragm	No	38.6/Titin 3.7	0	Normal	No effect	Hypothyreosis, RA
5 (23)	Norway	Probable	Generalized	No	8.0	NA	Normal	Not use	No

*UL, upper limb, LL, lower limb, NA, not applied, CMAP, compound muscle action potential, EMG, electromyogram, RA, rheumatoid arthritis; ALS, amyotrophic lateral sclerosis; AChR-ab, acetylcholine receptor antibodies; RNS, repetitive nerve stimulation; CT, computed tomography*.

Group 4: two clinically identified ALS patients presented with fluctuating ptosis and diplopia as initial symptoms followed by dysarthria, dysphagia, and limb muscle weakness, with normal concentration of sera AChR-ab and RNS test results. These patients were not responsive to cholinesterase inhibitors or immunosuppressive therapy (18). Evidence was considered insufficient to make the diagnosis of MG.

## Discussion

Herein, we presented two cases from our database and summarized the clinical characteristics of all the other cases that have been reported. Coexistence of ALS and MG is rare. However, Turner et al. (27) found that there were significantly more cases of ALS associated with a prior autoimmune disease, including MG. Association between two diseases may be driven by dysregulation of the immune system in both conditions. General and tissue-specific immune activation is seen in ALS (28). Deficiency or decrease of T-regulatory cells (Treg cells) (29, 30), upregulated atrophy-related atrogenes, and neuronal nitric oxide synthase abnormalities (31, 32) could be found in both MG and ALS. Immunoglobulin from ALS patients can affect neuromuscular junction functional characteristics (33), and the activity of AChRs also seems to play a role in the innervation and re-innervation of muscle fibers (28). Experimental evidence indicates that muscle and neuromuscular junctions may be sites of disease manifestation in a very early stage of ALS (34). According to the “dying-back” hypothesis, it is possible that neuromuscular transmission failure and postsynaptic membrane damage in MG patients may precede lower and upper motor neuron loss, thereby increasing the probability of developing ALS (28).

For patients who had ever been diagnosed with MG (as in Group 1), if their muscle weakness relapsed and/or progressed to more regions accompanied by upper motor neuron signs and was unresponsive to AChR inhibitors or immune-modulating therapy, it was not difficult to make an accurate diagnosis of ALS according to the EEC criteria (1). By comparing the localization of myasthenia symptoms and the site of onset of ALS in Group 1, we found that they were always at different regions except in three cases. In addition, muscle atrophy is a common sign of ALS but is relatively rare in MG patients. Therefore, when muscle weakness reappeared or was aggravated in affected muscles in different regions from prior MG or was accompanied by muscle wasting, reexamination of EMG should be recommended and a possible diagnosis of ALS should be taken into consideration. It has been reported that some bulbar onset MG with positive anti-AChR or anti-MuSK antibodies can mimic ALS (23, 35–37), so experimental immune-modulating therapy is necessary when it is too difficult to differentiate MG from ALS.

In the Italian case series, patients with ALS after MG had frequent bulbar onset of ALS symptoms (60%) (4) compared to 17% in a French study (22). The difference may come from bias due to a small number of cases. Taking all reported cases together, the proportion of bulbar onset ALS (36%, shown in Table 4) was consistent with epidemiological data of ordinary sporadic ALS (reported bulbar onset in 30–40% of ALS cases).

**Table 4:** **Summary of patients in Group 1 and Group 2**.

	Myasthenia gravis (MG) as inaugural disease	Amyotrophic lateral sclerosis (ALS) as inaugural disease	Total
Total no.	12	8	20
Male:female	8:4	6:2	14:6
Age at onset of MG (range)	59 (26–82)	60 (39–89)	59 (26–89)
Age at onset of ALS (range)	71 (55–83)	59 (34–89)	66 (34–89)
Interval between two conditions	3 months–41 years	0–5 years	0–41 years
**ALS site of onset**			
Limb, *n* (%)	8/12 (67%)	5/7 (71%)	13/19 (68%)
Bulbar, *n* (%)	4/12 (33%)	2/7 (29%)	6/19 (32%)
**Localization of MG symptoms**			
Ocular, *n* (%)	10/12 (83%)	7/8 (88%)	17/20 (85%)
Bulbar, *n* (%)	6/12 (50%)	5/8 (63%)	11/20 (55%)
Limbs, *n* (%)	4/12 (33%)	0/8 (0%)	4/20 (20%)

In Group 1, the mean age at onset of ALS was 71 years old, which is much older than that of patients in Group 2 (mean age of 59 years old, Table 4) or general sporadic ALS patients (reported mean age of 58–63 years old) (38). We speculate that immune-modulating therapy for prior MG at an early stage may have some protective or suppressive effect to delay the onset of motor neuron damage. Immune therapy trials have been unsuccessful in ALS (39, 40); however, timing is of great significance considering that ALS patients might be already in a late stage of the disease with irreparable motor neuron damage at the time of diagnosis. If we could identify ALS very early and initiate immune-modulating therapy at an asymptomatic stage, would it be effective? This possibility is supported by evidence that many molecular changes occur at the neuromuscular junctions at very early stages of ALS prior to symptom onset. By approaching therapeutic intervention from the three sides of the neuromuscular junction (the motor neuron, the muscle fiber and the terminal Schwann cells), the neuromuscular junction may be stimulated to remain in a healthy state for longer, and therefore, the onset of the disease might be more efficiently postponed (41). However, it should be noted that this is still speculation based on a clinical phenomenon, and the hypothesis needs to be verified by more well-established evidence.

As in Group 2, if ALS patients complain of fluctuating muscle weakness, concurrence of MG should be considered. RNS tests, assays of autoimmune antibodies related to the neuromuscular junction and experimental cholinesterase inhibitor treatment are useful methods for establishing diagnosis. As we can see in Group 2, the myasthenia symptoms mainly involved ocular and bulbar regions, presenting as ptosis, diplopia, dysphagia, dysarthria, and head drop, which might represent a phenotype prone to this association. Occurrence of ophthalmoplegia is rare in ALS; however, in those with advanced or bulbar onset ALS, it is likely that more extensive pathological changes in the brainstem give rise to supra-nuclear gaze palsy or slow saccades. When ophthalmoplegia occurs in prolonged or brainstem involved ALS without fluctuating phenomenon, fatigability, abnormal RNS and autoimmune antibodies, the diagnosis would not be myasthenia.

Acetylcholine receptor antibodies are considered to be highly specific for the diagnosis of MG, and these autoantibodies could also be found in 5% of ALS patients without clinical evidence of MG (42). One study traced the fluctuation of AChR-ab titers during the progression of ALS, and it seemed to increase during the periods when the patient’s ALS became more severe and decrease during periods when the patient was clinically more stable (17). The mechanism by which AChR-ab are produced in ALS cases is unknown. It may be related to the morphological alterations of neuromuscular junctions that are present at an early stage (43) due to autoimmune reaction against degenerative AChR at neuromuscular junctions. There were three patients with negative AChR-ab in Group 2, and two of them were positive for LRP4 antibodies. A recent study noted that LRP4 antibodies could be detected in 24 out of 104 ALS patients (23%) whose plasma was negative for AChR antibodies (44). Since LRP4 antibodies are found more frequently in ALS patients than in MG patients, they may have a direct pathogenic activity in the denervation process of ALS. The first patient we described was AChR-ab and MUsK-ab negative, but we did not test for LRP4 antibodies. It is worth noting that a compound muscle action potential decrease in an RNS test is not a specific phenomenon of MG, as it can also be found in 53% of typical ALS patients (45, 46). In ALS and MG overlap patients, an RNS test could also be negative, as for the last two patients in Table 2 (24). All patients in Group 2 were responsive to cholinesterase inhibitors, but patients in Groups 3 and 4 were not. Considering that fluctuating symptoms could be overlooked due to a marked weakness in ALS and that the specificity of the RNS test may be decreased, we recommend that in the context of ALS, the diagnosis of concomitant MG syndrome should thus be discussed only in the presence of suggestive clinical features (fluctuating symptoms and fatigability especially involved in ocular and/or bulbar regions) or autoimmune antibodies related to neuromuscular junction in addition to a positive response to cholinesterase inhibitors.

## Conclusion

The coexistence of ALS and MG is rare and requires thoughtful interpretation of clinical manifestations. These findings indicate a relationship between the two diseases and support the hypothesis that immunological mechanisms and alterations in the neuromuscular junction are related to ALS pathogenesis. Immune-modulating therapy at an early stage before onset of ALS symptoms might have protective effects on postponing motor neuron degeneration.

## Ethics Statement

This study was approved by the Ethics Committee of Clinical Research of Peking Union Medical College Hospital (Beijing, China), with written informed consent from all subjects whose medical records were reviewed.

## Author Contributions

HT: conception of the work, data acquisition and analysis, literature search, and writing of the first draft. LC: conception and organization of the work, manuscript review, and critique. YG, ML, XL, YH, JY, DS, DL, and FZ: clinical evaluation of patients, review, and critique.

## Conflict of Interest Statement

The authors declare that the research was conducted in the absence of any commercial or financial relationships that could be construed as a potential conflict of interest.
